# Lifelong dietary protein restriction induces denervation and skeletal muscle atrophy in mice

**DOI:** 10.1016/j.freeradbiomed.2024.09.005

**Published:** 2024-09-06

**Authors:** Ufuk Ersoy, Atilla Emre Altinpinar, Ioannis Kanakis, Moussira Alameddine, Anna Gioran, Niki Chondrogianni, Susan E. Ozanne, Mandy Jayne Peffers, Malcolm J. Jackson, Katarzyna Goljanek-Whysall, Aphrodite Vasilaki

**Affiliations:** a Department of Musculoskeletal and Ageing Science, Institute of Life Course & Medical Sciences (ILCaMS), The MRC - Versus Arthritis Centre for Integrated Research Into Musculoskeletal Ageing (CIMA), University of Liverpool, Liverpool, UK; b Chester Medical School, Faculty of Medicine and Life Sciences, University of Chester, Chester, UK; c Institute of Chemical Biology, National Hellenic Research Foundation, Athens, Greece; d MRC Metabolic Diseases Unit, Wellcome Trust-MRC Institute of Metabolic Science, Addenbrooke’s Treatment Centre, Addenbrooke’s Hospital, University of Cambridge Metabolic Research Laboratories, Cambridge, UK; e Department of Physiology, School of Medicine and REMEDI, CMNHS, University of Galway, Galway, Ireland

**Keywords:** Skeletal muscle, Atrophy, Protein restriction, Denervation, Proteostasis, Oxidative stress

## Abstract

As a widespread global issue, protein deficiency hinders development and optimal growth in offspring. Maternal low-protein diet influences the development of age-related diseases, including sarcopenia, by altering the epigenome and organ structure through potential increase in oxidative stress. However, the long-term effects of lactational protein restriction or postnatal lifelong protein restriction on the neuromuscular system have yet to be elucidated. Our results demonstrated that feeding a normal protein diet after lactational protein restriction did not have significant impacts on the neuromuscular system in later life. In contrast, a lifelong low-protein diet induced a denervation phenotype and led to demyelination in the sciatic nerve, along with an increase in the number of centralised nuclei and in the gene expression of atrogenes at 18 months of age, indicating an induced skeletal muscle atrophy. These changes were accompanied by an increase in proteasome activity in skeletal muscle, with no significant alterations in oxidative stress or mitochondrial dynamics markers in skeletal muscle later in life. Thus, lifelong protein restriction may induce skeletal muscle atrophy through changes in peripheral nerves and neuromuscular junctions, potentially contributing to the early onset or exaggeration of sarcopenia.

## 1 Introduction

It is well-established that dietary interventions can affect organismal health and lifespan [1]. One of the most broadly studied of these is calorie restriction, which increases the lifespan in species, including mice and rats [2,3]. While the role of macronutrients in dietary interventions is still under investigation, studies have reported that dietary protein restriction without a limitation in calorie consumption is associated with metabolic health and longevity in flies, mice, rats, and humans [4–8]. However, maternal protein restriction increased the risk of developing age-related diseases in their offspring, including cardiovascular diseases, type 2 diabetes, and sarcopenia [9]. This indicates that the timing and duration of protein restriction have a pivotal role in mediating organismal health. Richardson et al. (2021) reported that lifelong restriction of dietary branched-chain amino acids reduced frailty in mice [1]. However, we recently demonstrated that lifelong protein restriction starting from birth accelerates the loss of skeletal muscle fibres by impairing mitochondrial homeostasis and proteostasis [10].

Skeletal muscle motor units comprise a motor neuron and the myofibers it innervates. Neurophysiological alterations play a pivotal role in driving age-related skeletal muscle atrophy and fibre loss [11]. These changes consist of loss of the motor neuron and denervation-reinnervation cycle and induced impairments in the components of neuromuscular junctions (NMJ), including the reduced overlap of the pre-and postsynaptic structures and acetylcholine (ACh) receptors [12,13]. Strikingly, the age-related impairments in NMJs observed in rats occurred prior to myofiber atrophy [14]. NMJ stability is maintained by a sophisticated signalling pathway involving communication from the terminal motor neuron to the muscle fibre. A protein released from the terminal axon, agrin stabilises the assembly of NMJ by activating a muscle-specific protein kinase in postsynaptic ACh receptor known as muscle-associated receptor tyrosine kinase (MuSK). Muscle wasting occurs as a result of denervation or malfunctioning of signal transmission in NMJs, which is based on the regulation of specific signalling pathways, including the agrin-MuSK pathway [15]. Myelin is a sphingolipid-based multilayer sheath around the nerves generated by Schwann cells in the peripheral nervous system [16]. This sheath is formed by the spiral wrapping of the neuronal axon and facilitates the electrical impulse propagation resulting in an increase in the nerve conduction velocity [17]. There was no evidence indicating that demyelination directly caused skeletal muscle atrophy. However, autoimmune diseases of the peripheral nerves, including neuropathies, did result in muscle weakness through demyelination in humans [18]. A rat study reported that isocaloric severe maternal protein restriction (6 %) during gestation and lactation reduced the NMJ area in the soleus (SOL) muscle [19]. Another animal study demonstrated a protective effect of mild dietary restriction during adult life on the skeletal muscle and peripheral nerve in rats [20]. Taken together, the integrity of peripheral nerve and NMJs is crucial for skeletal muscle function and health; however, there is no data investigating the effects of long-term lactational protein restriction on muscle innervation and the examination of sciatic nerves and NMJs innervating the muscles.

Skeletal muscle is a highly dynamic organ that undergoes a striking transformation in response to environmental factors. Denervation led to skeletal muscle atrophy through altering mitochondria-mediated apoptosis via the Bcl-2-associated X (BAX)/B-cell lymphoma 2 (Bcl-2) pathway [21]. During muscle denervation, the BAX/Bcl-2 ratio was increased, resulting in the elevated release of cytochrome *c* from mitochondria to the cytosol [22]. Cytochrome *c* release from mitochondria stimulated apoptosis by recruiting and activating procaspase 9. This activation, in turn, induced the cleavage and activation of caspase-3 [23]. Caspase-3 stimulated the activity of proteasome in myotubes [24]. Denervation may, therefore, lead to the activation of proteasomal activity through mitochondria-regulated pathways. Moreover, sciatic nerve transection led to a reduction in mitochondrial number and activation of mitophagy in mice through the downregulation of mitofusin-1 (MFN1) [25]. Furthermore, the denervated muscle exhibited a disorganised mitochondrial network and increased production of reactive oxygen species from mitochondria [26,27]. Although our group reported that lifelong protein restriction reduced mitochondrial biogenesis, impaired AMP-activated protein kinase (AMPK) signalling, and disturbed proteolysis in mice [10], the effects of lifelong protein restriction on mitochondrial dynamics, proteasomal activities, and oxidative stress profile are yet to be elucidated.

Therefore, the aim of the present study was to determine the effect of lifelong protein restriction or the long-term effects of lactational protein restriction on the morphology of peripheral nerves, NMJs and skeletal muscles. We also investigated whether changes in the sciatic nerves and NMJs were accompanied by alterations in mitochondrial dynamic markers, oxidative stress markers or proteasome function in skeletal muscle. We demonstrated that a postnatal lifelong low-protein diet induced a denervation phenotype and led to demyelination in the sciatic nerve, along with the presence of centralised nuclei and an increase in the gene expression of atrogenes, suggesting induced skeletal muscle atrophy.

## 2 Materials and method

### 2.1 Animals

For this study, B6. Cg-Tg (Thy1-YFP)16Jrs/J mice (stock number 003709, Jackson Laboratory) were used. The mice were housed in individual vented cages with a fixed light cycle (21 ± 2 °C, 12-h light/dark cycle). All experiments received ethical approval from the University of Liverpool Animal Welfare Ethical Review Body (AWERB) and were performed in accordance with UK Home Office guidelines under the UK Animals (Scientific Procedures) Act 1986 (PPL numbers: P4A0493B2 and PP3047542).

### 2.2 Experimental design

**Fig. 1:**
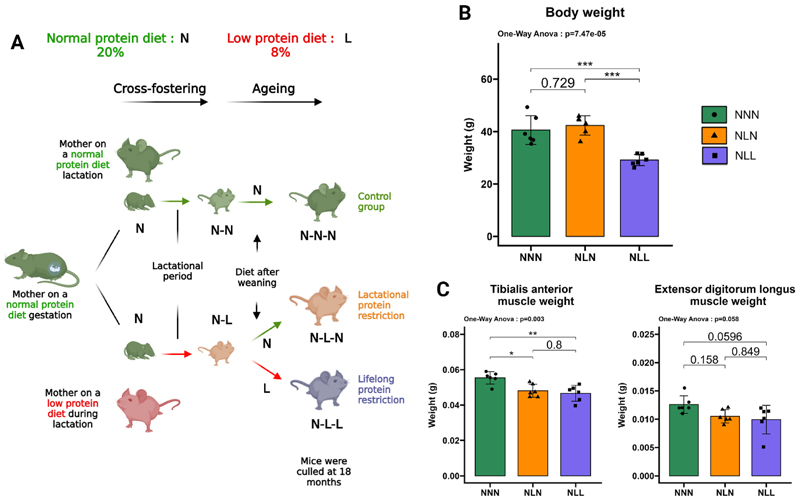
Experimental design, body weight, and muscle weights. (**A**) Normal-Normal-Normal (NNN) mice were provided with a normal protein diet both during pregnancy and after birth. Normal-Low-Normal (NLN) mice were nursed by dams on a low-protein diet during lactation and then weaned onto a normal protein. Normal-Low-Low (NLL) received a lifelong protein-restricted diet. (**B**) The body weights of 18-month-old mice in the NNN, NLN, and NLL groups, *n* = 6. (**C**) The weights (g) of TA and EDL muscles, *n* = 6. The results are presented as the mean ± standard deviation (mean ± SD). Statistical significance was set at *p < 0.05, **p < 0.01, ***p < 0.001, determined by ordinary one-way ANOVA followed by Tukey’s multiple comparisons test.

Two weeks before mating, nulliparous female mice were given either a normal-protein diet (N, 20 % crude protein; code 824,226, Special Diet Services, UK) or a low-protein diet (L, 8 % crude protein; code 824,248, Special Diet Services, UK) *ad libitum*. Male mice of the same age, which had been maintained on a normal-protein diet, were utilised for mating. Newborn pups were cross-fostered to dams fed either a low-protein diet or a normal-protein diet within 24 h of birth, generating Normal-Low (NL) and Normal-Normal (NN) groups of mice. NN mice were maintained on a normal-protein diet at the end of the weaning period (day 21), resulting in Normal-Normal-Normal (NNN) mice (n = 6). To observe the impacts of lifelong protein restriction, NL mice were weaned onto a low-protein diet and generated Normal-Low-Low (NLL) mice (n = 6). Additional NL mice were fed with a normal protein diet, creating Normal-Low-Normal (NLN) mice (n = 6). Mice were fed *ad libitum* water and food. Mice were sacrificed at 18 months (Fig. 1A). Following sacrifice and prior to dissection, body weights were recorded. Tibialis anterior (TA), EDL, and gastrocnemius (GAS) muscles were carefully dissected and weighed.

### 2.3 Histological analyses

Sciatic nerves were carefully dissected at the post-vertebral level and just before the pre-branching of the tibial and common fibular branches, with the removal of connective tissue surrounding the nerve. The nerves were fixed with 10 % Neutral Buffer Formalin (NBF, Thermo Fisher Scientific, Waltham, MA, USA) and washed with phosphate-buffered saline (PBS) with 0.01 % sodium azide and stored in 70 % ethanol at 4 °C until analysis. The day before cryosectioning, sciatic nerves were washed with PBS three times and kept in 30 % sucrose in PBS at 4 °C overnight. Nerves were placed in a cryomold and frozen in liquid nitrogen in Optimal Cutting Temperature (OCT – Thermo Fisher Scientific, Waltham, MA, US). Sections with a thickness of 10 μm were cut using a Leica CM1950 (Leica Biosystems, Newcastle upon Tyne, UK). Samples were air-dried for 30 min and washed with PBS- 0.5 % Tween 20 three times. Sections were again air-dried for 30 min and incubated with 0.2 % Triton X100 in PBS for 15 min at room temperature. The sections were blocked in 2 % bovine serum albumin (BSA) (Sigma-Aldrich Ltd, Gillingham, Dorset, United Kingdom), 15 % goat serum, and 0.25 % Triton X-100 in PBS. Cryosections were incubated with myelin basic protein (MBP – Abcam, ab40390) 1:250 in blocking buffer overnight at 4 °C and subsequently with Alexa Fluor 568 secondary fluorescent antibody (Invitrogen, Renfrewshire, UK) for an hour at room temperature. Following mounting with VECTASHIELD Antifade Mounting Medium (Vector Laboratories Ltd., Peterborough, UK), sections were left in a dark environment. A Zeiss LSM 800 confocal microscope (Zeiss, Ober-kochen, Germany) were used to visualise sections. The resulting images were segmented using Cellpose 2.0 (Supplementary Fig. 1), and the Regions of Interest (ROI) were analysed using ImageJ (U.S. National Institutes of Health, US) [28].

EDL muscles were carefully dissected, fixed in 10 % NBF, washed with PBS-Sodium azide and kept in 70 % ethanol at 4 °C until use. To allow the calculation of appropriate mouse numbers for each of the analyses, statistical power calculations were performed based on preliminary and published data. Three EDL muscles were randomly selected for this experiment. Samples were then washed with PBS and incubated with Bungarotoxin, Alexa Fluor™ 647 conjugate (1:500 - Invitrogen, Paisley, United Kingdom) for 30 min. This process was carried out in the dark at room temperature. Muscles from Thy1-YFP mice were imaged using a Nikon A1 microscope (Nikon, Kingston, UK). A Z-stack of images was taken, and a maximum projection was generated from the stack. The resulting images were analysed using the protocol outlined by Jones et al. [29], employing ImageJ.

### 2.4 Haematoxylin and eosin staining

GAS muscles were dissected and positioned vertically on cork discs prior to embedding in OCT (Thermo Fisher Scientific, Waltham, MA, US). Samples were submerged in liquid nitrogen-cooled isopentane (Sigma-Aldrich Ltd, Gillingham, Dorset, United Kingdom). A Leica CM1950 cryostat (Leica Biosystems, Newcastle upon Tyne, UK) was used to cut sections with a thickness of 10 μm. Following this, sections were air-dried for 30 min, fixed with 4 % paraformaldehyde (PFA) for 2 min, and washed twice with PBS for 5 min. Samples were submerged in Harry’s haematoxylin solution (Sigma-Aldrich Ltd, Gillingham, Dorset, United Kingdom) for 45 s, after which they were washed with running tap water for 30 s. Slides were then dipped into Eosin Y solution (Sigma-Aldrich Ltd, Gillingham, Dorset, United Kingdom) for 15 s and washed again with running tap water for 30 s. Sections were sequentially dehydrated in 70 % ethanol, 90 % ethanol, and 100 % ethanol for 30 s each and cleared with xylene for 30 s. Finally, sections were left to air dry and mounted with Histomount mounting media (National Diagnostics, Charlotte, NC, US). Images were captured using a Nikon Ci (Nikon, Kingston, UK). The resulting images were analysed in ImageJ using the cell counting tool as described previously [30]. Manual analysis of central nuclei was conducted, and more than 150 fibres were counted per experimental sample.

### 2.5 Gene expression analyses

GAS muscles were carefully dissected, snap-frozen in liquid nitrogen, and stored at – 80 °C until analysis. Frozen GAS muscles were powdered using a pestle and mortar in liquid nitrogen. RNA extraction from the powdered GAS muscles was undertaken using TRIzol (Thermo Fisher Scientific, Waltham, MA, US) and purified with RNeasy mini kits (Qiagen, Manchester, UK) according to the manufacturer’s instructions. To eliminate the risk of genomic contamination, extracted RNA was treated with DNase (Qiagen, Manchester, UK). 1000 ng RNA was utilised to synthesise cDNA by the High-Capacity cDNA Reverse Transcription Kit (Applied Biosystems, Warrington, UK). qPCR was conducted with the Meridian Bioscience SensiMix SYBR Hi-ROX Kit (Scientific Laboratory Supplies, Nottingham, UK) with reaction volumes of 20 μl. Rotor-Gene Q Series software (Qiagen, Manchester, UK) was used for data analysis. mRNA expression levels were normalised to the expression of 18S rRNA, which was stable in our experiment (Supplementary Fig. 2). The delta-delta Ct method (2^–ΔΔCt^) was undertaken to calculate relative gene expression levels [31]. Primer pairs for atrogin-1 MuRF1, MuSK and 18S were validated prior to the analysis and are listed in Supplementary Table 1.

### 2.6 Western blotting

RIPA Lysis Buffer (Sigma, Poole, UK) containing an EDTA-free protease and phosphatase inhibitor (Thermo Fisher Scientific, Waltham, MA, US) was added to a portion of the ground GAS muscle to extract total protein. Homogenised samples were sonicated twice for 30 s, followed by centrifugation for 10 min at 14,000×*g* at 4 °C. The protein concentration of the collected supernatant was determined using a BCA Protein Assay (Sigma, Poole, UK). Gel electrophoresis was carried out using NuPAGE™ 4–12 %, Bis-Tris, 1.0–1.5 mm, Mini Protein Gels (Invitrogen, Renfrewshire, UK). The separated proteins were subsequently transferred to a polyvinylidene fluoride (PVDF) membrane. For blocking, membranes were incubated in 5 % BSA (Sigma, Poole, UK) in Tris-buffered saline (TBS) for an hour at room temperature. Primary antibody conditions were optimised prior to experiments. Membranes were then treated with the antibody overnight at 4 °C. Following incubation with Licor secondary antibodies (Licor, Bad Homburg, Germany), the blots were imaged with the Licor Odyssey CLx. Band densities were analysed with ImageJ and normalised to Ponceau staining or GAPDH (ab8245, 1:2000) [32].

Protein carbonyls were detected using an OxiSelect™ Protein Carbonyl Immunoblot Kit (Cell Biolabs, San Diego, California, United States), which derivatises proteins directly on the membrane following protein separation by SDS-PAGE and transfer. PVDF membranes were used and derivatised as per the manufacturer’s instructions. The membrane was blocked with 5 % BSA (Sigma, Poole, UK) in TBS-Tween 20 for 1 h at room temperature and incubated with primary antibody against Dinitrophenyl (DNP) (Cell Biolabs 1:1000 dilution in 5 % BSA TBS-T) overnight at 4 °C. Following incubation with Licor secondary antibodies (Licor, Bad Homburg, Germany), the blots were imaged with the Licor Odyssey CLx. Band densities were analysed with ImageJ. Subsequently, the membrane was stripped, washed, blocked, and incubated with primary antibody against GAPDH. Results then were normalised to the GAPDH intensity.

### 2.7 Antibodies

The primary antibodies used are indicated below, along with their corresponding dilution factors. Anti-Myeloperoxidase (Abcam, Cambridge, UK - ab208670 1:1000), Anti-DRP1 (Cell Signaling, Danvers, Massachusetts, United States - D6C7, 1:1000), Anti-phospho-DRP1^Ser616^ (Cell Signaling, 3455, 1:500), Anti-OPA1 (Cell Signaling, 80471, 1:1000), Anti-MFN1 (Abcam, ab126575, 1:700), Anti-BAX (Cell Signaling, 2772, 1:500), Anti-Bcl-2 (Cell Signaling, 3498, 1:500), Anti-caspase-3 (Cell Signaling, 9662, 1:500), Anti-TRX2 (Abcam, ab185544, 1:2000), Anti-NOX4 (Abcam, 133303, 1:500), Anti-GPX1 (Abcam, ab22604, 1:1000), Anti-PRX3 (Abcam, ab73349, 1:1000), Anti-3-nitrotyrosine (3-NTs; Abcam, ab61392, 1:1000).

### 2.8 Proteasome activity assay

The degradation rate of the fluorogenic substrate Suc-Leu-Leu-Val-Tyr-AMC (Bachem, Bubendorf, Switzerland) was measured to assess the chymotrypsin-like activity of 20S in an ATP-depleted manner and of 26S activity in an ATP-dependent manner, as described previously [33–35]. Briefly, a part of GAS muscle was lysed through freeze-thaw cycles (liquid nitrogen and 37 °C water bath) in a lysis buffer containing 25 mM HEPES, 250 mM sucrose, 1 mM EDTA, 10 mM magnesium chloride, and 1.7 mM dithiothreitol (DTT). To deplete ATP for 20S proteasome activity, 0.1 mg/ml hexokinase (Sigma-Aldrich Ltd, Gillingham, Dorset, United Kingdom) and 15 mM 2-deoxyglucose were added to the incubation buffer containing 225 mM Tris base, 45 mM potassium chloride, 7.5 mM magnesium acetate, 7.5 mM magnesium chloride, and 1 mM DTT. To measure the 26S proteasome activity, ATP with 2 mM final concentration was introduced into the incubation buffer. The release of 7-Amino-4-methylcoumarin (AMC) from suc-LLVY-AMC was measured with a setting of λex = 360 nm and λem = 485 nm, with a 3-min interval read for 120 min at 37 °C. To ensure specificity, the assays were repeated in the presence of lactacystin (Enzo Life Sciences, Farmingdale, New York, US), a specific proteasome inhibitor [36]. Free AMC was used as a standard to calculate enzymatic activity. Protein extracts used for activity assay were run on a NuPAGE™ 4–12 %, Bis-Tris gel and Coomassie blue staining (Sigma-Aldrich Ltd, Gillingham, Dorset, United Kingdom) was used for the loading control.

### 2.9 Electron microscopy

Snap-frozen TA muscles were immediately submerged in 4 % paraformaldehyde in 0.1 M sodium cacodylate buffer (pH 7.4) on a rotator at room temperature for 20 min. To allow the calculation of appropriate mouse numbers for each of the analyses, statistical power calculations were performed based on preliminary and published data. Three TA muscles were randomly selected for this experiment. Samples were then subjected to immersion in a solution containing 2.5 % glutaraldehyde in 0.1M sodium cacodylate buffer (pH 7.4) for a further 60 min.

Tissues were rinsed with 0.1 M sodium cacodylate buffer and then stained with 2 % osmium tetroxide, followed by 1.5 % potassium ferrocyanide. The samples were then incubated overnight with aqueous 1 % Uranyl acetate. The final stain was with Walton’s lead aspartate (0.02 M lead nitrate in 0.03 M L-aspartic acid, adjusted to pH 5.5) for 30 min at room temperature. Staining steps were carried out in a Pelco Biowave®Pro (Ted Pella Inc., Redding, CA, US), for 1 min, with a minimum of 3 × 5 min washes with ddH2O.

Samples were dehydrated in a graded ethanol series, starting with 30 %, followed by 50 %, 70 %, and 90 % in ddH2O for 5 min each. This was followed by immersion in 100 % ethanol and 100 % acetone, both for 3 × 5 min. The samples were infiltrated with Agar 100 Hard Premix resin (Agar Scientific, Stansted Essex, UK) at a 1:1 ratio (resin: 100 % acetone), followed by 3 sets of 30-min resin-only infiltration. Tissues were then embedded in rubber moulds and cured for 48 h at 60 °C.

To prepare the blocks, tissue was trimmed on an ultramicrotome and viewed using a TM 4000 desktop SEM (Hitachi, Tokyo, Japan) to check for correct orientation before sectioning onto formvar coated copper grids (Agar Scientific, Stansted Essex, UK). Sections were viewed in a Tecnai T12 transmission electron microscope (FEI Company, Hillsboro, OR) at 120 KV, fitted with a Rio 16 digital camera (Gatan, Pleasanton, CA).

### 2.10 Statistical analysis

n *refers to the number of mouse pups. R version 4.2.2 was used for data analysis, and data are presented as mean* ± *standard deviation (mean* ± *SD) with* n = *3*–*6. One-way ANOVA and Tukey’s multiple comparison test were used for statistical comparisons. NNN mice were considered as the control group. Normal distribution was checked with Shapiro-Wilk normality test, and p < 0.05 of confidence was considered to indicate statistical significance*.

## 3 Results

### 3.1 Muscle weights and body weights

To investigate the long-term effects of lactational protein restriction, newborn litters were nursed by low-protein-fed mothers during lactation and weaned onto a normal protein diet (NLN) (Fig. 1A). These mice had a similar body weight, TA muscle weight, and EDL muscle weight compared to the control group (NNN) at 18 months of age (Fig. 1B and C). To reveal the impact of lifelong protein restriction, newborn litters were nursed by a mother on a low-protein diet during lactation and fed by a low-protein diet after weaning (NLL) (Fig. 1A). These mice exhibited significantly smaller body weight and reduced TA muscle weight, with slightly reduced EDL muscle weight compared to the NNN mice at 18 months of age (Fig. 1B and C). Moreover, our laboratory previously showed that GAS muscle weight was significantly lower in NLL mice at 3 months of age compared to the control group, but only a slight reduction was observed at 18 months of age [10].

Since our previous findings demonstrated that lifelong dietary protein restriction accelerated the loss of type II fibres, we investigated potential changes in the NMJs of EDL muscles, conducted histological analyses, western blotting, and proteasomal activity assays in the GAS muscle, and performed TEM in the TA muscle. We chose to focus our current investigation on the GAS, TA, and EDL muscles, which predominantly consist of type IIB and IIDB fibres in mice [37].

### 3.2 Lifelong protein restriction induced morphological and structural changes in sciatic nerve and NMJs

**Fig. 2:**
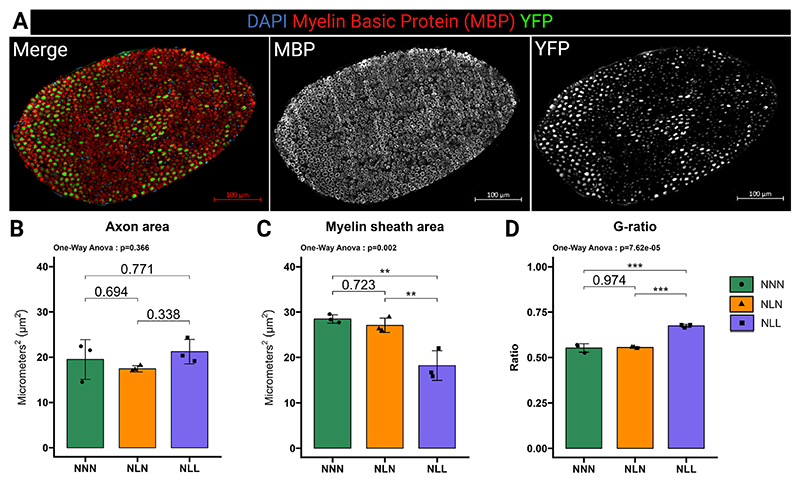
Lifelong protein restriction reduced myelin sheath in the sciatic nerve. (**A**) Representative confocal images of MBP (red), YFP (green), and DAPI in sciatic nerve from 18-month-old mice. Scale bar, 100 μm. (**B**) Axon area of sciatic nerve from 18-month-old mice. At least 1000 axons were measured per nerve, *n* = 3. (**C**) Lifelong protein restriction reduced myelin sheath area of the sciatic nerve in 18-month-old mice. At least 1000 axons were measured per nerve, *n* = 3. (**D**) The G-ratio, the ratio between axon diameter and the total nerve diameter (axon and myelin), *n* = 3. The results are presented as the mean ± standard deviation (mean ± SD). Statistical significance was set at *p < 0.05, **p < 0.01, ***p < 0.001, determined by ordinary one-way ANOVA followed by Tukey’s multiple comparisons test.

Ageing is associated with selective denervation of fast twitch fibres, along with a decrease in the number of myelinated neuronal axons within these muscle fibres [38]. Since we previously reported that life-long protein restriction led to an acceleration in the loss of type II fibre and a reduction in muscle fibre size [10]. We hypothesised that a lifelong low-protein diet would negatively impact the myelinisation in the peripheral nerve. To investigate this, we used sciatic nerve sections from Thy1/YFP mice to measure myelin sheath thickness and analysed more than 1000 axons per experimental sample. We demonstrated that the myelin sheath area was significantly reduced in NLL mice, while the axon area remained similar compared to the control group (Fig. 2A, B, C). The G-ratio, the ratio between axon diameter and the total nerve diameter (axon and myelin), is reversely associated with the speed of conduction. This ratio was significantly increased in NLL mice relative to the NNN mice (Fig. 2D), suggesting a slower conduction speed. However, there were no differences observed in myelin sheath area, axon area, and G-ratio between NLN and the control mice (Fig. 2B, C, D).

**Fig. 3:**
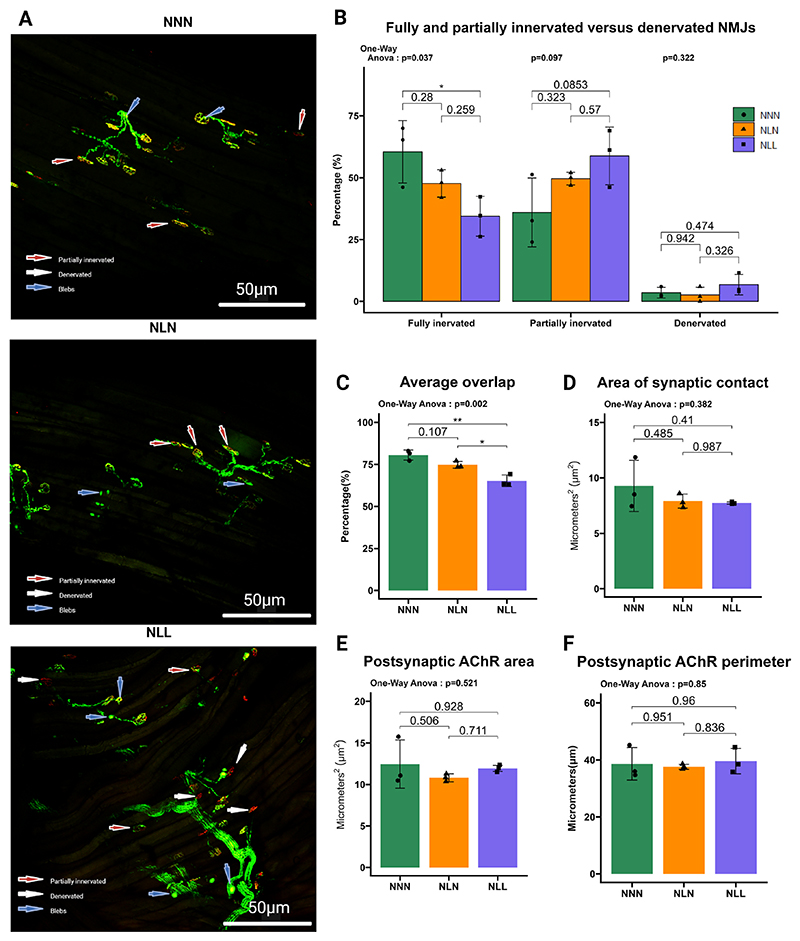
Lifelong protein induced a denervation phenotype in EDL muscle. (**A**) Representative confocal images of heterogeneous distribution of innervated and denervated neuromuscular junction on the surface of EDL muscle from Thy1-YFP NNN, NLN, and NLL mice at 18 months of age, *n* = 3. (**B)** The illustrations demonstrate the percentages of fully innervated (80–100 % overlap), partially innervated (15–79 % overlap), and denervated (<15 % overlap) NMJS and (**C**) the average overlap of presynaptic nerve terminal and postsynaptic motor endplate on the surface of EDL muscle from NNN, NLN, and NLL mice, *n* = 3. (**D, E, F**) Analysis of (D) the area of synaptic contact, (E) the post-synaptic ACh receptors area, and (F) the post-synaptic ACh receptors perimeter of EDL muscles from NNN, NLN, and NLL mice, *n* = 3. The results are presented as the mean ± standard deviation (mean ± SD). Statistical significance was set at *p < 0.05, **p < 0.01, ***p < 0.001, determined by ordinary one-way ANOVA followed by Tukey’s multiple comparisons test.

Changes in the neuromuscular system have a great impact on skeletal muscle loss as reduction in motor unit number is secondary to motor neuron loss [11]. Examination and analysis of at least 50 NMJs on the surface fibres of EDL muscles demonstrated that the percentage of fully innervated (80–100 % overlap) NMJs was significantly reduced, while the percentages of partially innervated (15–79 % overlap), and denervated (<15 % overlap) NMJs slightly increased in NLL mice compared to the control group (Fig. 3 A and 3B). Moreover, these mice exhibited a significant decrease in the average overlap of the presynaptic nerve terminal and postsynaptic motor endplate (Fig. 3C). Analysis of the area of synaptic (Fig. 3D), the post-synaptic ACh receptors area (Fig. 3E), and the post-synaptic ACh receptors perimeter (Fig. 3F) demonstrated no significant difference between NLL and NNN mice. However, the percentage of fully innervated, partially innervated, and denervated NMJs (Fig. 3B), the average overlap of presynaptic nerve terminal and post-synaptic motor endplate (Fig. 3C), the area of synaptic contact (Fig. 3D) and the area and perimeter of the post-synaptic ACh receptors (Fig. 3E and F) in NLN mice were unchanged compared to the control group.

Together, these results show that lifelong protein restriction induced demyelination in the sciatic nerve and a denervation phenotype in skeletal muscle. However, lactational protein restriction had no significant effects on sciatic nerve myelinisation and innervation of skeletal muscle in later life.

### 3.3 Lifelong protein restriction induced skeletal muscle atrophy

**Fig. 4:**
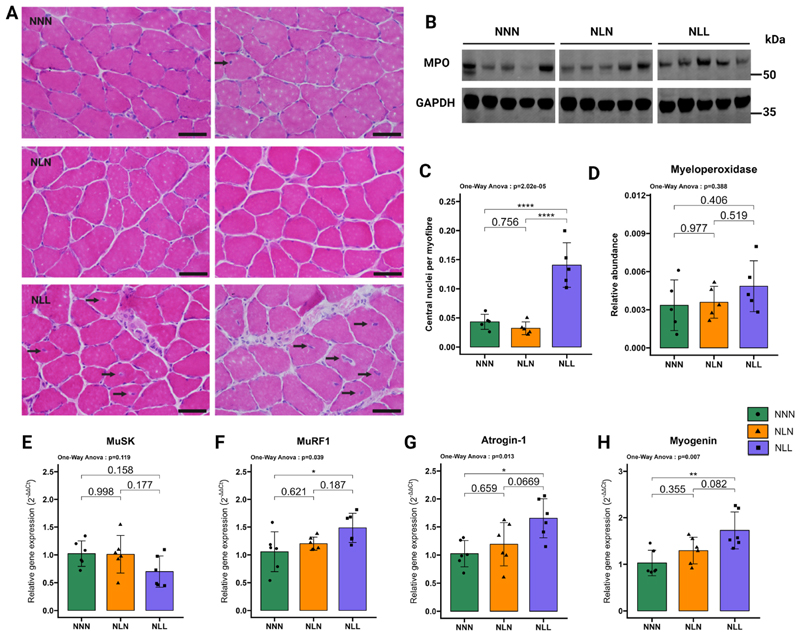
Lifelong protein restriction led to atrophy in GAS muscle. (**A, C**) Representative H&E staining of GAS muscle. NLL muscles were characterised by central nucleation, indicated by arrows (scale bar: 300 μm). (C) Lifelong protein restriction significantly increased central nuclei per myofibre ration in GAS muscle. At least 150 myofibres were counted per experimental sample, *n* = 5. (**B, D**) Western blot analysis of myeloperoxidase (MPO). The samples were all simultaneously run on a single gel, and the images were cropped solely for inclusion in this figure. Either ponceau staining or GAPDH was utilised as the loading control, n = 5. (**E, F, G, H**) qPCR analysis of muscle-associated receptor tyrosine kinase (MuSK), Muscle RING-finger protein-1 (MURF1), Atrogin-1, and Myogenin in GAS muscles from NNN, NLN, and NLL mice at 18 months of age, *n* = 5–6. The results are presented as the mean ± standard deviation (mean ± SD). Statistical significance was set at *p < 0.05, **p < 0.01, ***p < 0.001, determined by ordinary one-way ANOVA followed by Tukey’s multiple comparisons test.

Centralised nuclei are routinely found in regenerating muscle fibres and certain muscle disorders. Examination of haematoxylin and eosin (H&E)-stained muscle sections showed NLL mice exhibited a significant increase in central nuclei per myofibre ratio in their GAS muscle as well as an accumulation of non-contractile tissue (Fig. 4A–C). Macrophages translocate to the regenerating skeletal muscles and play a crucial role in the repair process [39]. As a heme-containing peroxidase, myeloperoxidase (MPO) is primarily expressed in neutrophils, monocytes, and macrophages. We found no significant changes in MPO protein abundance in NLL mice relative to the NNN mice (Fig. 4B–D). Postsynaptic ACh receptor MusK is necessary for the maintenance and formation of the NMJ [11]. We hypothesised that the changes observed in NMJs, and the sciatic nerve would be accompanied with the changes in genes in agrin-MuSK assembly. MuSK mRNA expression was slightly reduced in NLL mice compared with the control group (Fig. 4E). Surgical denervation has been demonstrated to lead to an upregulation in the markers known as atrogenes, including atrogin-1 and Muscle RING-finger protein-1 (MuRF1), which are molecular biomarkers of skeletal muscle atrophy [40,41]. Moreover, myogenin controls neurogenic atrophy and upregulates expression levels of MuRF1 and atrogin-1 following denervation [42]. We observed that MuRF1, atrogin-1, and myogenin gene expressions were significantly increased in NLL mice relative to the control group (Fig. 4 F, G, H). Thus, these results suggested a lifelong low-protein diet induced skeletal muscle atrophy.

It was reported that lactational protein restriction had negative impacts on skeletal muscle weight in mice in early life [43]. Here, we assessed the long-term effects of lactational protein restriction and observed no significant changes in the mRNA expression levels of MuSK (Fig. 4E), MuRF1 (Fig. 4F), atrogin-1 (Fig. 4G), and myogenin (Fig. 4H), nor in the protein abundance of MPO (Fig. 4B–D), in NLN mice compared to NNN mice at 18 months of age.

### 3.4 Lifelong protein restriction increased ATP-independent 26S proteasome activity in skeletal muscle

**Fig. 5:**
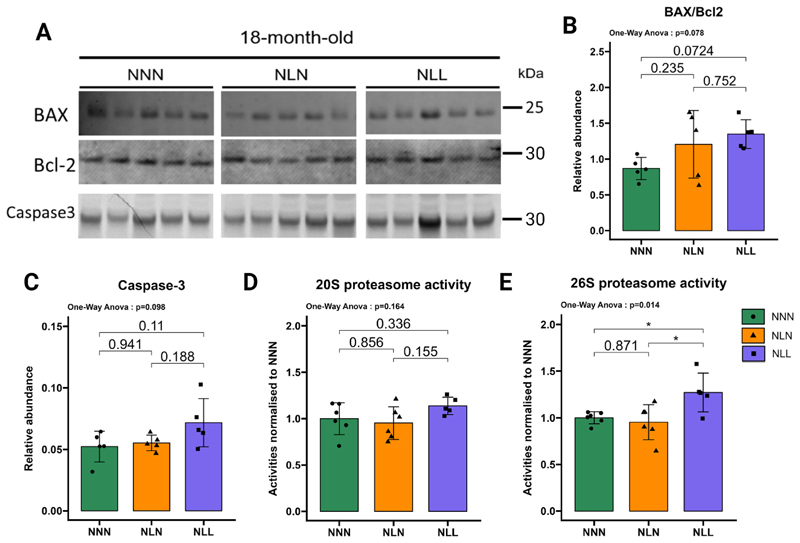
Lifelong protein restriction induced 26S proteasome activity in GAS muscle. (**A**) Western blot analysis of Bcl-2-associated X (BAX), B-cell lymphoma 2 (Bcl-2), and caspase-3 in GAS muscle. The samples were all run simultaneously on a single gel, and the images were cropped solely for inclusion in this figure. Ponceau staining was utilised as the loading control, *n* = 5. Images are provided in the source data file (**B**) The ratio of BAX/Bcl2 protein abundance. *n* = 5. (**C**) Quantification of caspase-3 protein abundance levels, *n* = 5. (**D**) 20S proteasome activity (μmol/s/mg protein) in GAS muscle. Samples were run on a gel and Coomassie blue staining was used as loading control. Activities normalised to NNN mice, *n* = 5–6. (**E**) 26S proteasome activity (μmol/s/mg protein) is increased in NLL mice compared to the control group. Samples were loaded on a single gel and Coomassie blue staining was used as loading control. Activities normalised to NNN mice, *n* = 5–6. The results are presented as the mean ± standard deviation (mean ± SD). Statistical significance was set at *p < 0.05, **p < 0.01, ***p < 0.001, determined by ordinary one-way ANOVA followed by Tukey’s multiple comparisons test.

Changes in proteasomal activities have been shown in cases of muscle atrophy or denervation [44]. BAX/Bcl-2-caspase-3 signalling pathway has regulatory control over proteasomal activities [23,24]. This led us to investigate the BAX/Bcl-2-caspase-3 signalling pathway and proteasome activity. We found no significant change in the BAX/Bcl-2 ratio in GAS muscle from NLL mice compared with the control group (Fig. 5A and B). Additionally, caspase-3 protein abundance was similar in NLL mice relative to the control group in their GAS muscle (Fig. 5A–C). An increase in chymotrypsin-like (CT-L) proteasome activity was reported in the case of muscle atrophy [45,46]. Therefore, we measured CT-L of 20S proteasome (ATP-independent degradation) and 26S proteasome (ATP-dependent degradation) in GAS muscle, and there was no significant difference in the 20S proteasome activity (CT-L) between NLL and NNN mice (Fig. 5D). However, the 26S proteasome activity (CT-L) was significantly increased in NLL mice (Fig. 5E), suggesting a lifelong low-protein diet altered 26S CT-L proteasome activity in skeletal muscle.

There was no significant impact of lactational protein restriction on the ratio of BAX/Bcl-2 (Fig. 5A and B) and caspase-3 protein abundance (Fig. 5A–C). Consistent with these results, the 20S proteasome activity (Figs. 5D) and 26S proteasome activity (Fig. 5E) were similar in NLN mice relative to the control group.

### 3.5 The effects of lifelong protein restriction on mitochondrial dynamics and mitochondrial redox markers in skeletal muscle

**Fig. 6:**
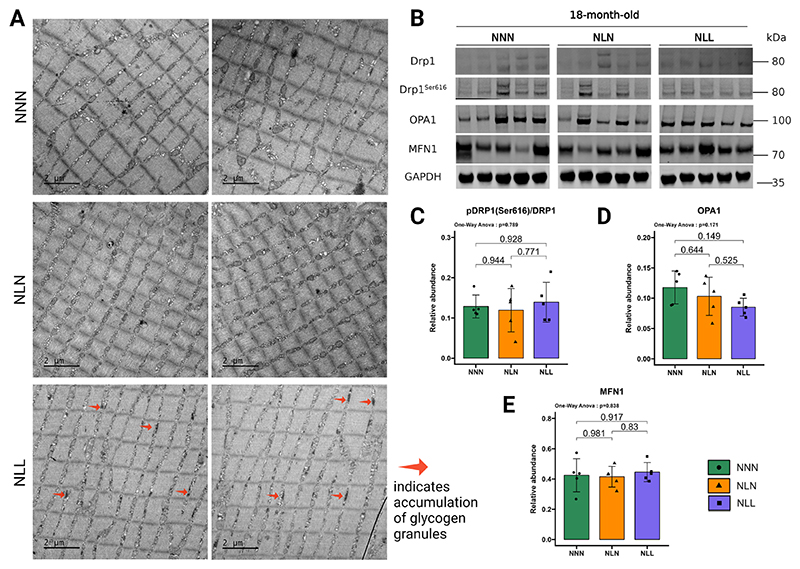
The effects of lifelong protein restriction or lactational protein restriction on mitochondrial dynamics in TA and GAS muscles (**A**) Representative transmission electron microscopy (TEM) images from transverse sections of TA muscles from 18-month-old NNN, NLN, and NLL mice. The arrows indicate the accumulation of glycogen granules. (**B**) Western blotting of dynamin-related protein 1 (DRP1), DRP1^Ser616^, Optic atrophy-1 (OPA1), and mitofusin-1 (MFN1). (**C**) Ratio of DRP1^Ser616^/DRP1 protein abundance. (**D, E**) Quantification of (D) OPA1 and (E) MFN1 protein abundance levels. The samples were all run simultaneously on a single gel, and the images were cropped solely for inclusion in this figure. Either ponceau staining or GAPDH was utilised as the loading control, *n* = 5. Images are provided in the source data file. The results are presented as the mean ± standard deviation (mean ± SD). Statistical significance was set at *p < 0.05, **p < 0.01, ***p < 0.001, determined by ordinary one-way ANOVA followed by Tukey’s multiple comparisons test.

Denervation-induced changes in the mitochondrial dynamics of denervated muscles [47]. MFN1 plays a pivotal role in mediating denervation-driven mitochondrial disorder [25]. NLL mice did not exhibit a significant difference in the protein abundance of MFN1 in their GAS muscle compared to the control group (Fig. 6B–E). Optic atrophy-1 (OPA1) and dynamin-related protein 1 (DRP1) are other GTPases that regulate mitochondrial fusion and fission [48]. Similar to MFN1, there were no significant differences in OPA1 and DRP1 protein abundance between NLL mice and NNN mice (Fig. 6B–D). Ser616 phosphorylation induces the translocation of DRP1 from the cytosol to the outer membrane of mitochondria, which is required for mitochondrial fission [49]. The ratio of DRP1^Ser616^/DRP1 was similar between NLL and NNN mice (Fig. 6C). Additionally, the abundances of mitochondrial dynamic proteins MFN1 (Fig. 6B–E), OPA1 (Fig. 6B–D), and DRP1 (Fig. 6B) were not significantly altered in NLN mice compared to the NNN mice. The DRP1^Ser616^/DRP1 ratio (Fig. 6C) was unchanged between NLN mice and the control group. To further assess the effects of the denervation phenotype on the mitochondrial shape and size in skeletal muscle, we used TEM to analyse the mitochondria of TA muscle (Fig. 6A). Observation of TEM images suggested that mitochondria from NLL mice were similar to those from NNN mice in terms of shape. Together, these data suggest that the denervation phenotype induced by lifelong protein restriction did not significantly alter mitochondrial dynamics in skeletal muscle.

Mitochondria and glycogen play crucial roles in metabolism and have increasingly been associated with various signalling pathways [50]. Studies have shown the age-related accumulation of glycogen granules in various tissues [51–54]. In the present study, we observed that lifelong protein restriction led to the accumulation of glycogen granules around the mitochondria in skeletal muscle (Fig. 6A).

**Fig. 7:**
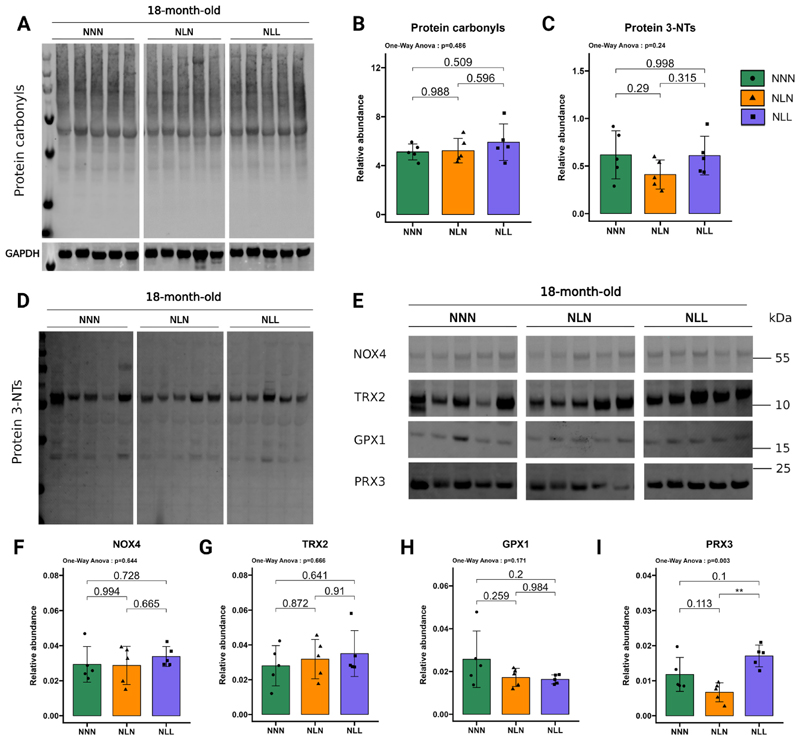
The effects of lifelong protein restriction or lactational protein restriction on markers of oxidative damage and antioxidant enzymes in GAS muscle (**A, B**) (A) Western blotting and (B) quantification of protein carbonyl. (**C, D**) (D) Western blotting and (C) quantification of protein 3-NTs. (**E**) Western blotting of NADPH oxidase 4 (NOX4), thioredoxin 2 (TRX2), glutathione peroxidase 1 (GPX1), and peroxiredoxin 3 (PRX3) in GAS muscle from 18-month-old NNN, NLN, and NLL mice. (**F, G, H, I**) Quantification of (F) NOX4, (G) TRX2, (H) GPX1, and (I) PRX3 protein abundance levels. The samples were all run simultaneously on a single gel, and the images were cropped solely for inclusion in this figure. Either ponceau staining or GAPDH was utilised as the loading control, *n* = 5. Images are provided in the source data file. The results are presented as the mean ± standard deviation (mean ± SD). Statistical significance was set at *p < 0.05, **p < 0.01, ***p < 0.001, determined by ordinary one-way ANOVA followed by Tukey’s multiple comparisons test.

Denervation-induced atrophy in skeletal muscle is linked to increased ROS production from mitochondria [55]. As we showed that lifelong protein restriction led to a partial denervation phenotype, we next assessed the protein abundance of mitochondria-located NADPH oxidase 4 (NOX4), which contributes to mitochondrial ROS production [56]. NOX4 protein abundance was not significantly different between NLL and NNN mice (Fig. 7E and F). Additionally, we investigated mitochondria-located antioxidant enzymes peroxiredoxin 3 (PRX3), thioredoxin 2 (TRX2), and glutathione peroxidase 1 (GPX1). NLL and NNN mice had similar TRX2 (Fig. 7G), GPX1 (Fig. 7H) and PRX3 (Fig. 7I) protein content. We also examined whether lifelong protein restriction led to increased protein oxidation in skeletal muscle. Consistent with the lack of changes in the 20S proteasome activity that has been shown to be responsible for the degradation of carbonylated proteins [57,58], western blot analyses for protein carbonyls indicated that there were no differences in GAS muscle in NLL mice compared to NNN mice (Fig. 7A and B). We also observed no significant differences between NLL and NNN mice in protein 3-NT levels in GAS skeletal muscle (Fig. 7C and D). Moreover, NLN mice demonstrated comparable NOX4 (Fig. 7E and F), TRX2 (Fig. 7E–G), GPX1 (Fig. 7E–G), and PRX3 (Fig. 7E–I) protein abundances to NNN mice. Western blot analysis of protein 3-NTs (Fig. 7A and B) and protein carbonyls (Fig. 7C and D) also indicated no significant differences between NLN and NNN mice at 18 months of age. Together, these findings suggest that a lifelong low-protein diet or lactational protein restriction did not result in changes in oxidative stress markers in skeletal muscle in later life.

## 4 Discussion

Dietary interventions are widely acknowledged to play a significant role in shaping organismal health and lifespan [1]. In this study, we demonstrated that lifelong dietary protein restriction induced a significant decrease in the area of axonal myelin sheaths and the percentage of fully innervated muscle fibres in male mice. These changes were accompanied by an increase in proteasome activity in skeletal muscle, with no significant alterations in oxidative stress or mitochondrial dynamics markers in skeletal muscle later in life. However, feeding a normal protein diet after lactational protein restriction did not have significant effects on the sciatic nerve or NMJs in skeletal muscle in male mice at 18 months of age compared with the control group.

Age-related decline in skeletal muscle mass greatly impacts whole-body metabolism and leads to sarcopenia and frailty, which are risk factors for adverse outcomes, including falls and loss of strength [59, 60]. This decline primarily results from skeletal muscle atrophy and fibre loss and is associated with motor neuron loss and neuromuscular junction instability [61]. These factors not only lead to a decline in strength and power but also impair coordination, collectively contributing to adverse outcomes [11]. It has been reported that approximately one billion people suffer from chronically insufficient protein intake [62–64]. As a widespread global issue, protein deficiency hinders development and optimal growth in offspring [65]. Our laboratory recently reported that a lifelong low-protein diet leads to accelerated loss of type IIa fibres and a reduction in muscle fibre size in later life by dysregulating ribosomal gene expression, autophagy, AMP-activated protein kinase (AMPK) signalling and mitochondrial homeostasis [10]. In the present study, we demonstrated that a postnatal lifelong low-protein diet induced a denervation phenotype and led to demyelination in the sciatic nerve. These changes were accompanied by alterations in NMJ components, including a slight reduction in MUSK1 mRNA expression and a significant increase in the expression levels of the myogenin, MuRF1 and atrogin-1, along with significant increase in the centralised nuclei per myofibre ratio in muscles containing predominantly type II fibres. Moreover, our laboratory recently reported that mice subjected to lifelong protein restriction exhibited a significantly accelerated decline in hanging time between 18 and 24 months of age [66]. In line with our findings, mice receiving a lifelong severe low-protein diet (5 %) exhibited reduced muscle force, morphological abnormalities, branching defects, and increased site fragmentation at the NMJ sites in EDL muscle, along with an increase in atrogin-1 gene expression level in TA muscle at 12 weeks of age [67]. Rats born to dams on a low protein diet (8 %) and maintained on the same diet throughout postnatal life exhibited increased protein and mRNA levels of atrogin-1 and mRNA level of MuRF1 in the GAS muscle at 16 weeks of age [68]. Taken together, lifelong protein restriction induces skeletal muscle atrophy through changes in peripheral nerves and NMJs in muscle consisting mainly of type II fibres. Age-related muscle atrophy is often fibre-type specific, with type II fibres being more susceptible to atrophy [69]. Therefore, these results suggest that a lifelong low-protein diet may potentially, contribute to the early onset or exaggeration of sarcopenia.

Skeletal muscle mass is highly dependent on protein synthesis and protein degradation regulated by the ubiquitin-proteasome system (UPS) and autophagy, which maintain a balanced and functional proteome [70]. The proteasome system is activated during most catabolic processes that lead to muscle atrophy [71]. For example, an increase in the 26S proteasome activity in skeletal muscle was found in the case of diabetes, acidosis, and denervation atrophy in rats [72–74]. The 20S proteasome activity was shown to be responsible for the selective removal of oxidised proteins [57]. In the present study, we demonstrated that feeding a postnatal lifelong low-protein diet increased 26S proteasomal activity in skeletal muscle. However, mice fed a lifelong protein diet had no significant changes in the activity level of the 20S proteasome, consistent with the lack of differences in oxidative stress markers. Although there were no statistically significant changes in the mitochondrial BAX/Bcl2 signalling pathway, we observed a trend indicating a potential increase in the BAX/Bcl2 ratio and elevated caspase-3 protein abundance in later life. Moreover, the increase in 26S proteasomal activity was accompanied by a significant rise in the expression levels of atrogenes MuRF1 and Atrogin-1, which are considered as contributors to muscle atrophy [75]. In line with our findings, an increase in total protein degradation, caspase-12 protein abundance, BAX protein abundance, and the BAX/Bcl-2 ratio was reported in rats that received prenatal and postnatal protein restriction at 16 weeks of age [68]. In addition to these results, we previously reported that lifelong protein restriction led to changes in other members of proteostasis, including impaired autophagy and reduced ribosomal gene expression in the GAS muscle at 18 months of age [10]. Thus, these findings collectively suggest that lifelong protein restriction induced skeletal muscle atrophy by impairing key members of the proteostasis network at the molecular level.

The immunoproteasome has been strongly implicated in the oxidative stress response and in aging within muscle tissue [76]. While modulation of the immunoproteasome is possible, this study focused only on the 20S and 26S proteasomes. Therefore, although we cannot rule out the involvement of this proteasome subtype, we did not detect any differences in the levels of carbonylated proteins or in general oxidative stress markers in our samples at 18 months of age (Fig. 7) that could suggest immunoproteasome induction. This finding could imply that at this age, the immunoproteasome is not yet responsive. Further experimentation at later stages would be valuable to determine whether a lifelong protein diet might induce the immunoproteasome beyond 18 months of age.

Denervation can lead to changes in the mitochondrial dynamics and redox homeostasis in skeletal muscles [47,55]. In the present study, we investigated the markers of mitochondrial dynamics and oxidative stress in the partially denervated skeletal from mice on a lifelong low-protein diet, and our findings revealed no changes in these markers. However, it is reported that the atrophy of fully denervated muscle is mediated by miR-142a-5p/MFN1, wherein changes in mitochondrial dynamics play a pivotal role downstream at two weeks post-operation [25]. Another study showed that the removal of a 5–10 mm section from the sciatic nerve led to a reduction in Mfn2 gene expression at day 5 and day 30 post-operation [47]. It was also demonstrated that denervated muscle had a greater susceptibility to mitochondria-mediated apoptosis, and this was greater in denervated muscle [21]. Moreover, it was reported that the denervation of skeletal muscle induced an increase in the release of hydrogen peroxide production from muscle mitochondria at day 7 post-denervation [77]. Interestingly, Scalabrin et al. [55] identified no changes in protein carbonylation and NOX4 and SOD2 protein contents in fully denervated skeletal muscle, while NADPH gp91phox and p67phox protein abundance were increased at day 14 post-operation. Key differences from our study include the extent of denervation; while previous studies have primarily focused on the impacts of complete denervation on mitochondrial dynamics and redox homeostasis, our research focused on the unique aspects of partial denervation induced by a lifelong protein diet. Future studies are needed to investigate whether a lifelong protein diet would lead to further denervation beyond 18 months of age once the sarcopenic characteristics have fully emerged.

It is well understood that maternal nutrition has a significant impact on the health outcomes of the offspring in later life. We observed no effects of lactational protein restriction on the sciatic nerve, NMJ, mitochondrial dynamics, and markers of oxidative stress. Longitudinal human and animal studies have indicated that a protein-deficient diet during pregnancy has negative impacts on the development, lifespan, and key regulatory signalling pathways [78–80]. However, Ozanne and Hales reported that the offspring from mothers on a low-protein diet during lactation had greater longevity [79]. We recently reported that the detrimental effects of lactational protein restriction observed in skeletal muscle during early life and adulthood can be corrected in later life by feeding a normal protein diet [10]. Therefore, the current data underscores that the timing of maternal nutritional programming has great impact in regulating the long-lasting impacts on the offspring.

Limitations of the study: 1) Changes in atrogenes, MuRF1, atrogin-1 and myogenin were investigated at the mRNA level, which does not always correspond to differences at the protein level for these markers. 2) Our study was limited to the analysis of TEM images. More comprehensive methods are essential for performing quantitative analyses of TEM images to identify the mitochondrial number, glycogen granules, and mitochondrial morphology.

In conclusion, the present study demonstrated lifelong protein restriction may lead to skeletal muscle atrophy through impairments in proteostasis at the molecular level, potentially driven by denervation-induced changes. This highlights the importance of proteostasis as a significant factor in skeletal muscle atrophy induced by prolonged protein restriction. Despite a considerable amount of evidence reporting the adverse effects of maternal undernutrition in early life, the data presented here indicate that the long-term effects of lactational protein restriction can be corrected in later life. Future studies focusing on the effects of low protein diet in old age, and in particular when a sarcopenic phenotype has fully developed (approximately 22–24 months of age in mice), could provide valuable insights into how diet influences muscle mass loss and musculoskeletal function in older individuals. This understanding could, in turn, lead to the development of effective interventions.

## Supplementary Material

Highlights

Supplementary Material
