# Supplementary material for: Lifelong dietary protein restriction induces denervation and skeletal muscle atrophy in mice
Source: Free Radic Biol Med. Author manuscript; Available in PMC 2025 Jan 14. (PMC7617303; doi:10.1016/j.freeradbiomed.2024.09.005)
Supplement: Highlights [file EMS202146-supplement-Highlights.docx]

**Highlights**

- Lifelong protein restriction induces skeletal muscle atrophy
- Lifelong protein restriction induces denervation in skeletal muscle
- Lifelong protein restriction impairs proteostasis in skeletal muscle
- Long-lasting effects of maternal nutritional programming depends on its timing
