# Supplementary material for: Lifelong dietary protein restriction induces denervation and skeletal muscle atrophy in mice
Source: Free Radic Biol Med. Author manuscript; Available in PMC 2025 Jan 14. (PMC7617303; doi:10.1016/j.freeradbiomed.2024.09.005)
Supplement: Supplementary Material [file EMS202146-supplement-Supplementary_Material.zip › 1-s2.0-S0891584924006488-mmc1.docx]

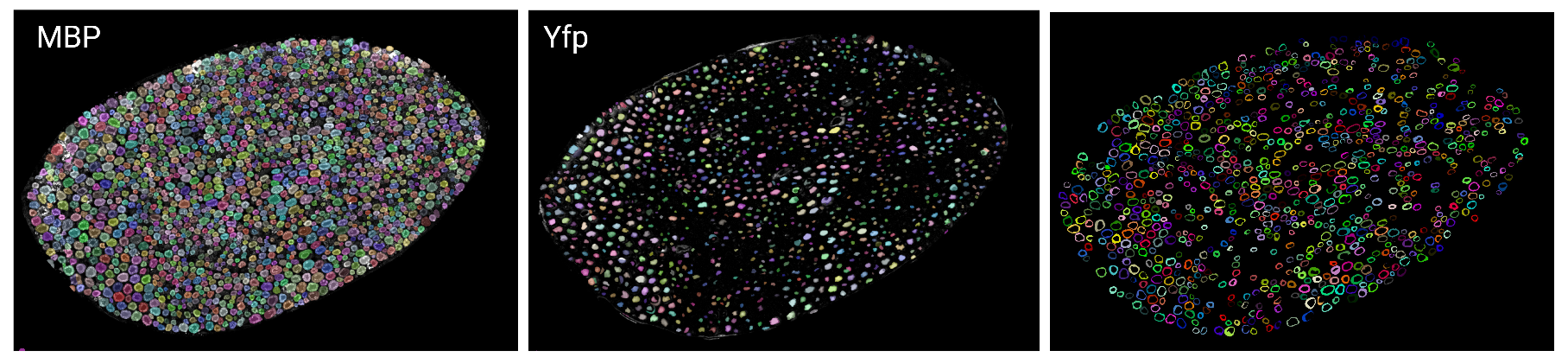


**Supplementary Figure 1. Illustration of measurement of myelin sheath area**. Images were segmented using Cellpose 2.0, and the resulting Regions of Interest (ROIs) were analyzed with ImageJ (U.S. National Institutes of Health, USA) as follows: first, (**a**) ROIs for axons and myelin sheath combined were selected, then (**b**) ROIs for axons only were extracted from the combined ROIs. (**c**) Remaining myelin rings were selected, and areas were calculated using BoneJ plugins.


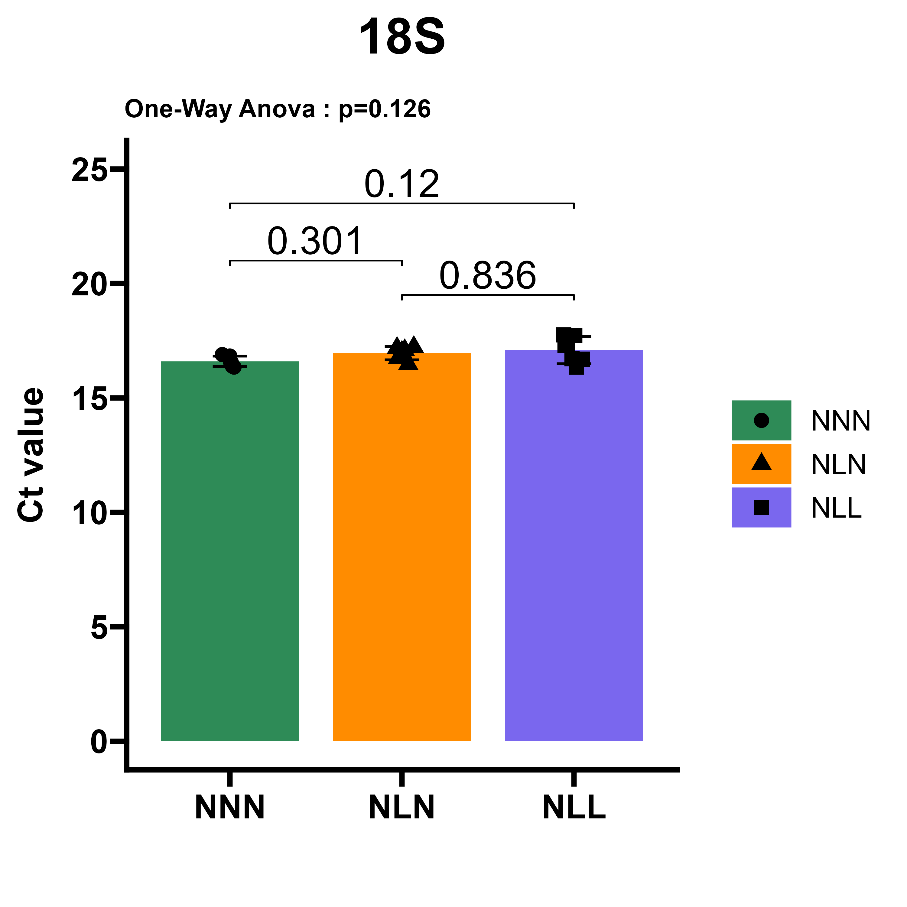


**Supplementary Figure 2. Expression level of 18S rRNA reference genes did not differ between groups. (a)** 18S Ct values in GAS skeletal muscle from 18-month-old NNN, NLN, and NLL mice. Results are expressed as the mean ± standard deviation (mean ± SD). Statistical comparisons were performed using ordinary one-way ANOVA with a Tukey’s multiple comparisons test, considering NNN as the control group.

**Supplementary Table 1** List of primers. Atrogin-1, Muscle RING-finger protein-1 (MuRF1), muscle associated receptor tyrosine kinase (MuSK), and 18S rRNA.

| *Gene* | Forward sequence | Reverse sequence | Exon-exon spanning | Primer efficiency | Validation |
| --- | --- | --- | --- | --- | --- |
| *Atrogin-1* | GCAGAGAGTCGGCAAGTC | CAGGTCGGTGATCGTGAG | + | 92.0% | + |
| *MuRF1* | AACGACCTCCAGACATGGAC | GACAGTCGCATTTCAAAGCA | + | 89.6% | + |
| *MuSK* | CCCTCCTCCGTGGTTTTCTA | GCTGTCTTTCTGTGACTTACTCC | + | 90.1% | + |
| *18S* | GGAAAGCAGACATCGACCTCA | AGTTCTCCAGCCCTCTTGGT | - | 95.2% | + |
