# Supplementary figures and images for: Lifelong dietary protein restriction induces denervation and skeletal muscle atrophy in mice
Source: Free Radic Biol Med. Author manuscript; Available in PMC 2025 Jan 14. (PMC7617303; doi:10.1016/j.freeradbiomed.2024.09.005)

## Slide 1
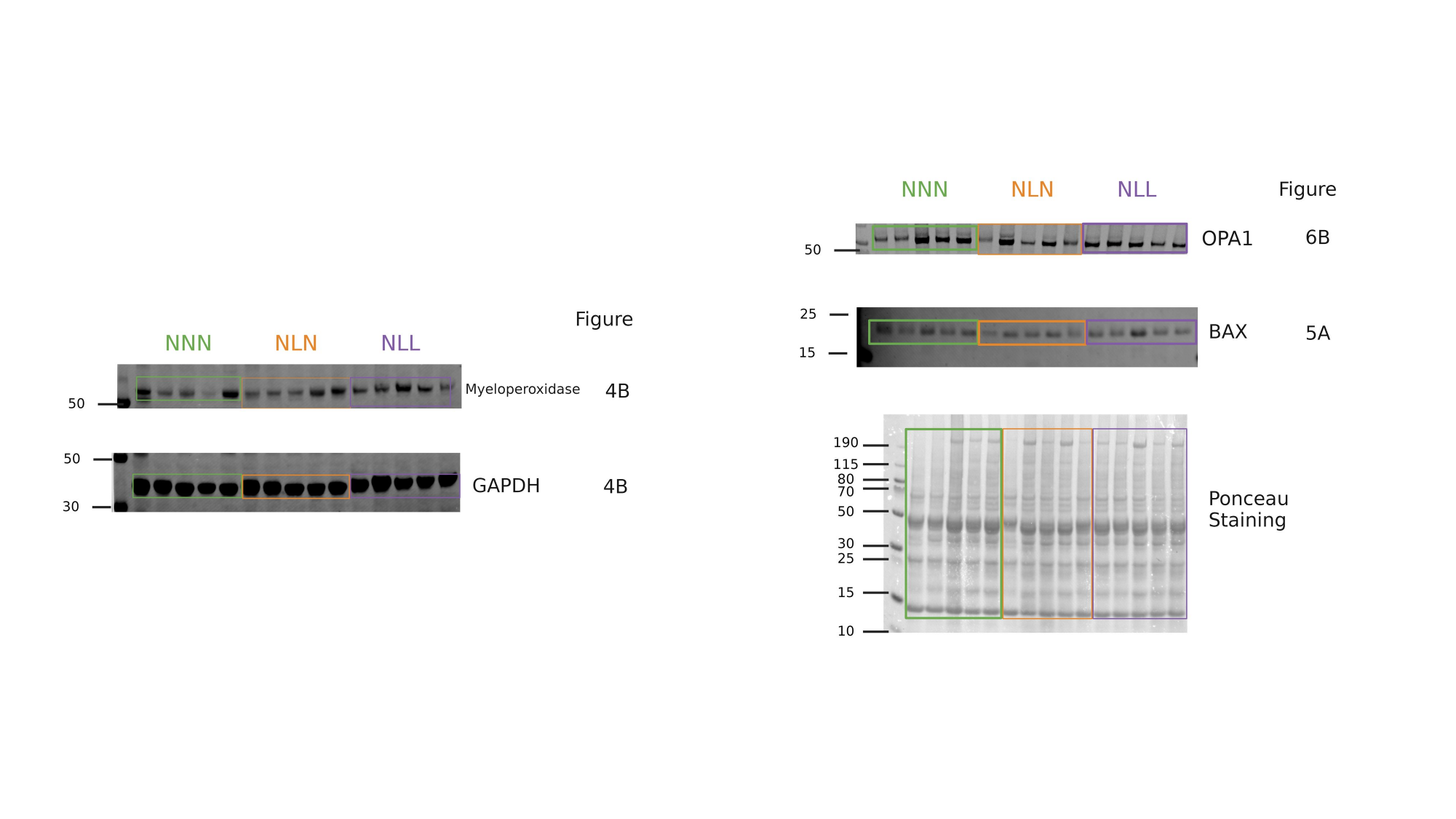

## Slide 2
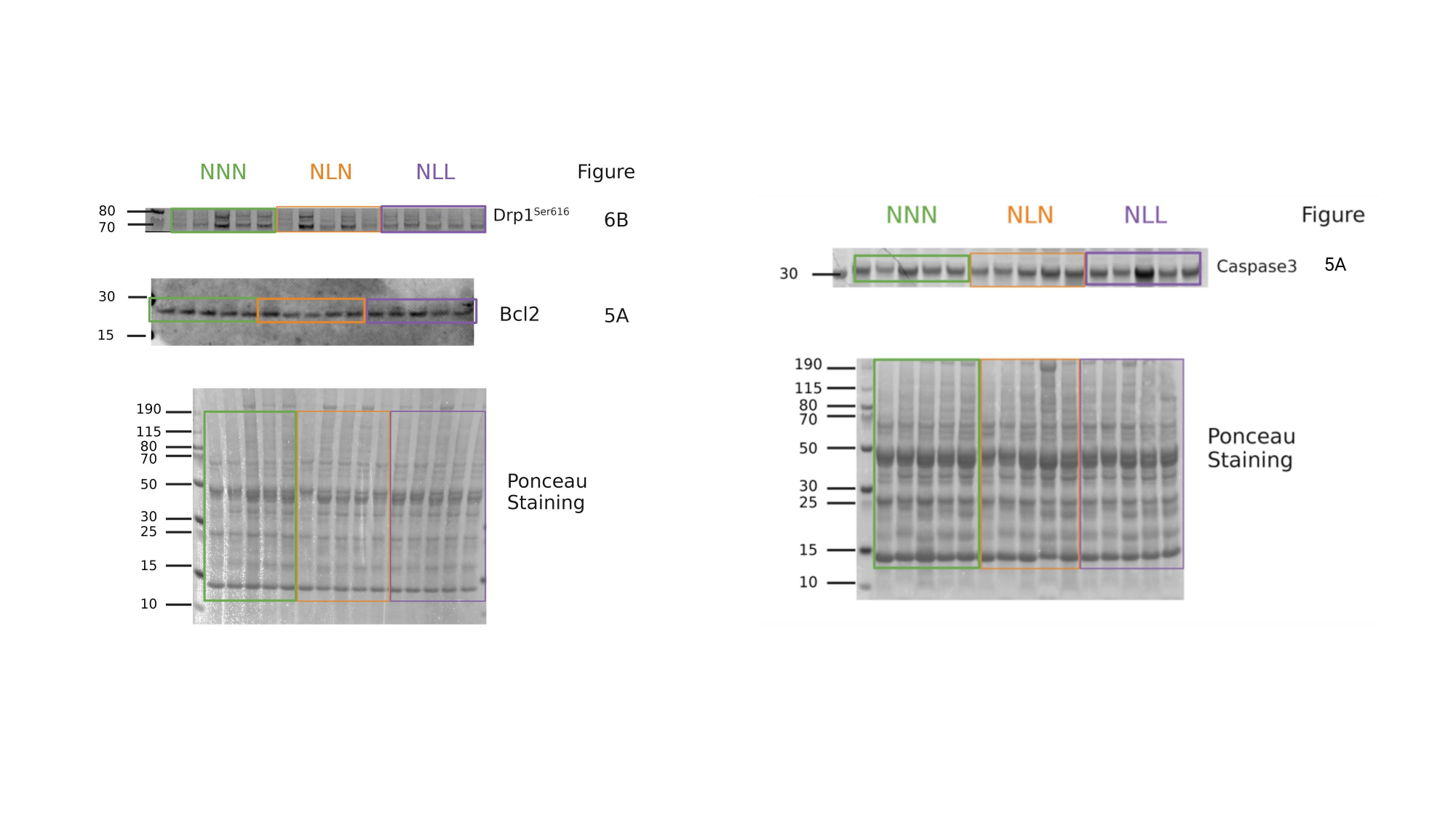

## Slide 3
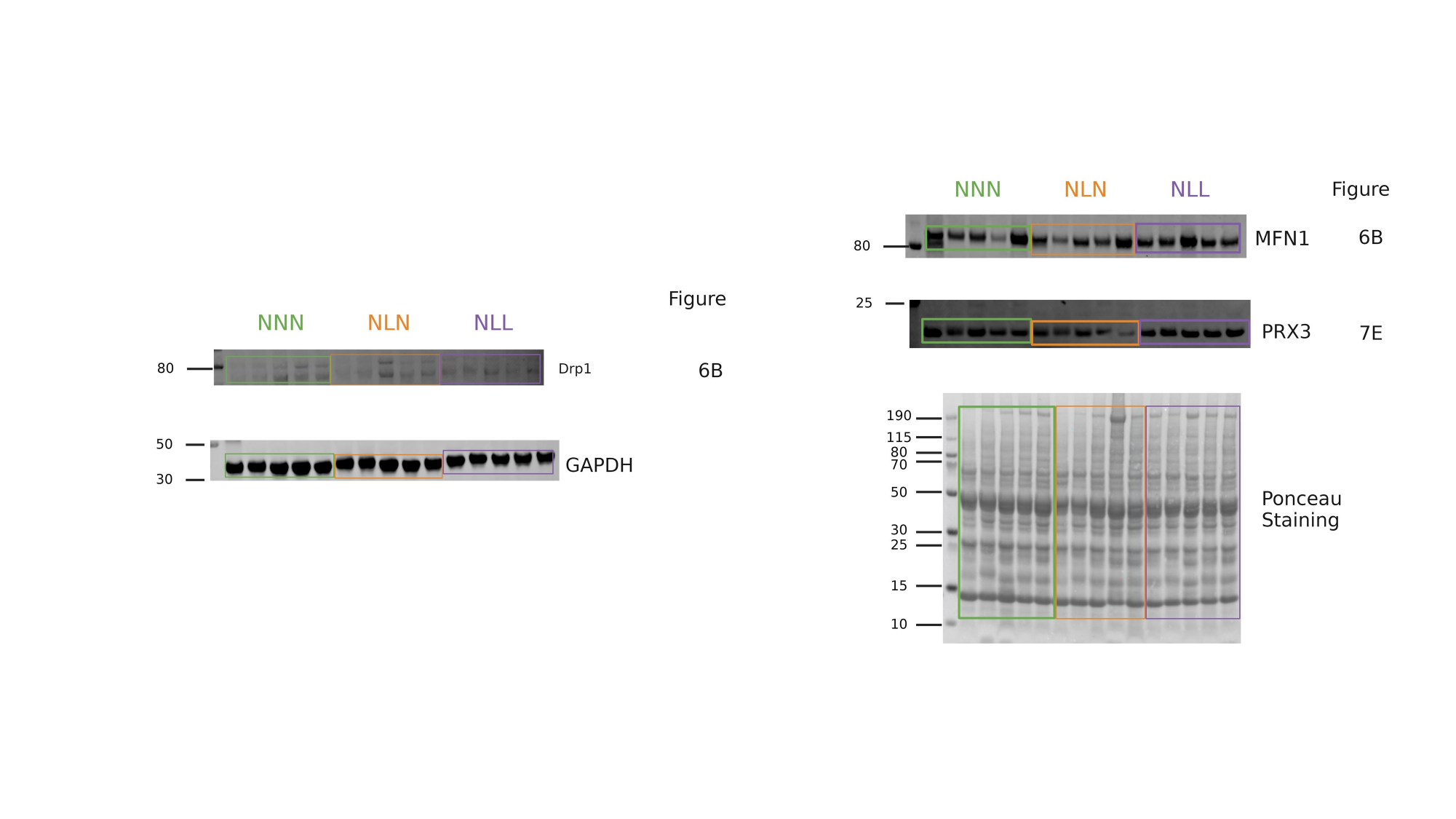

## Slide 4
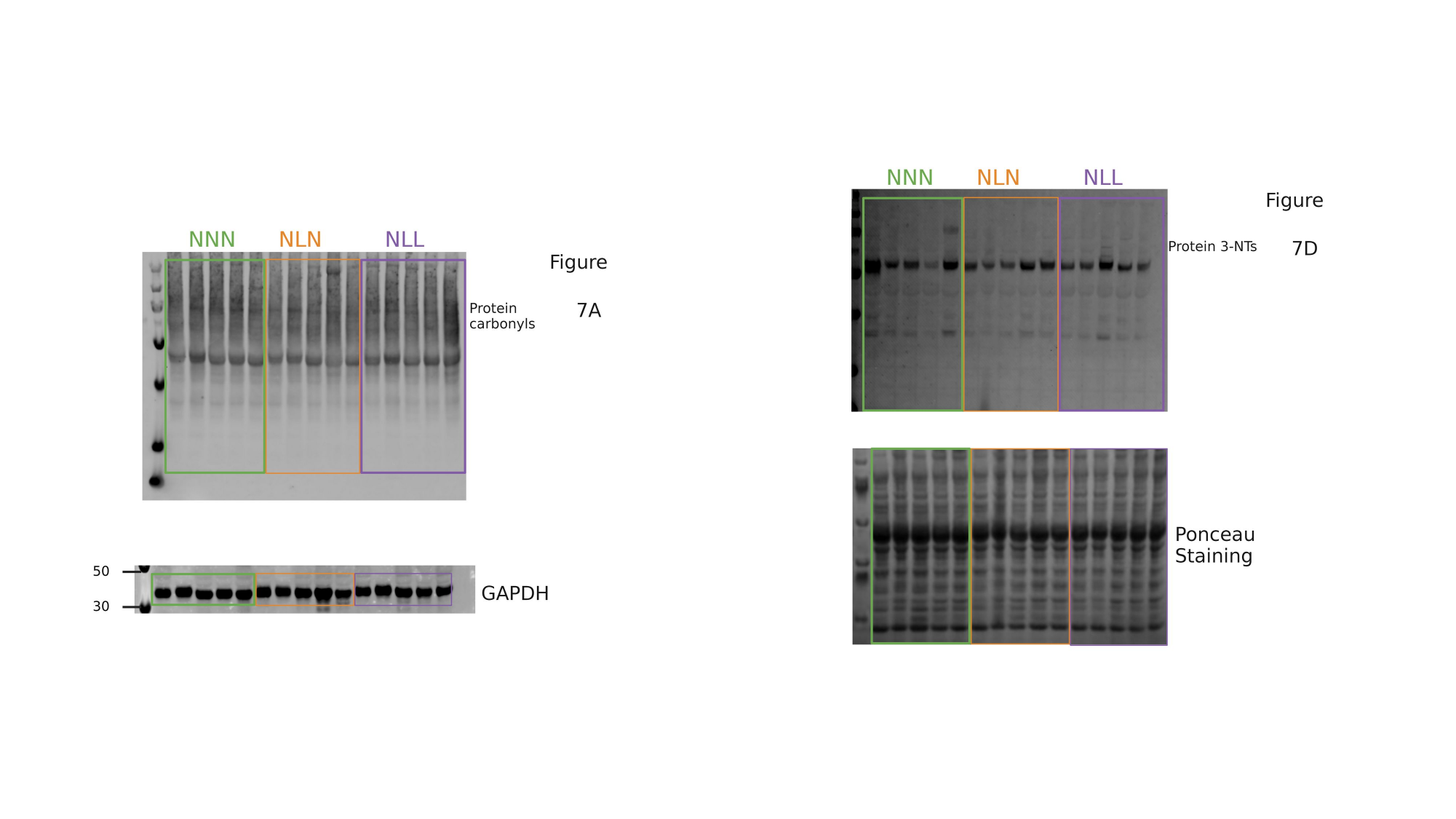

## Slide 5
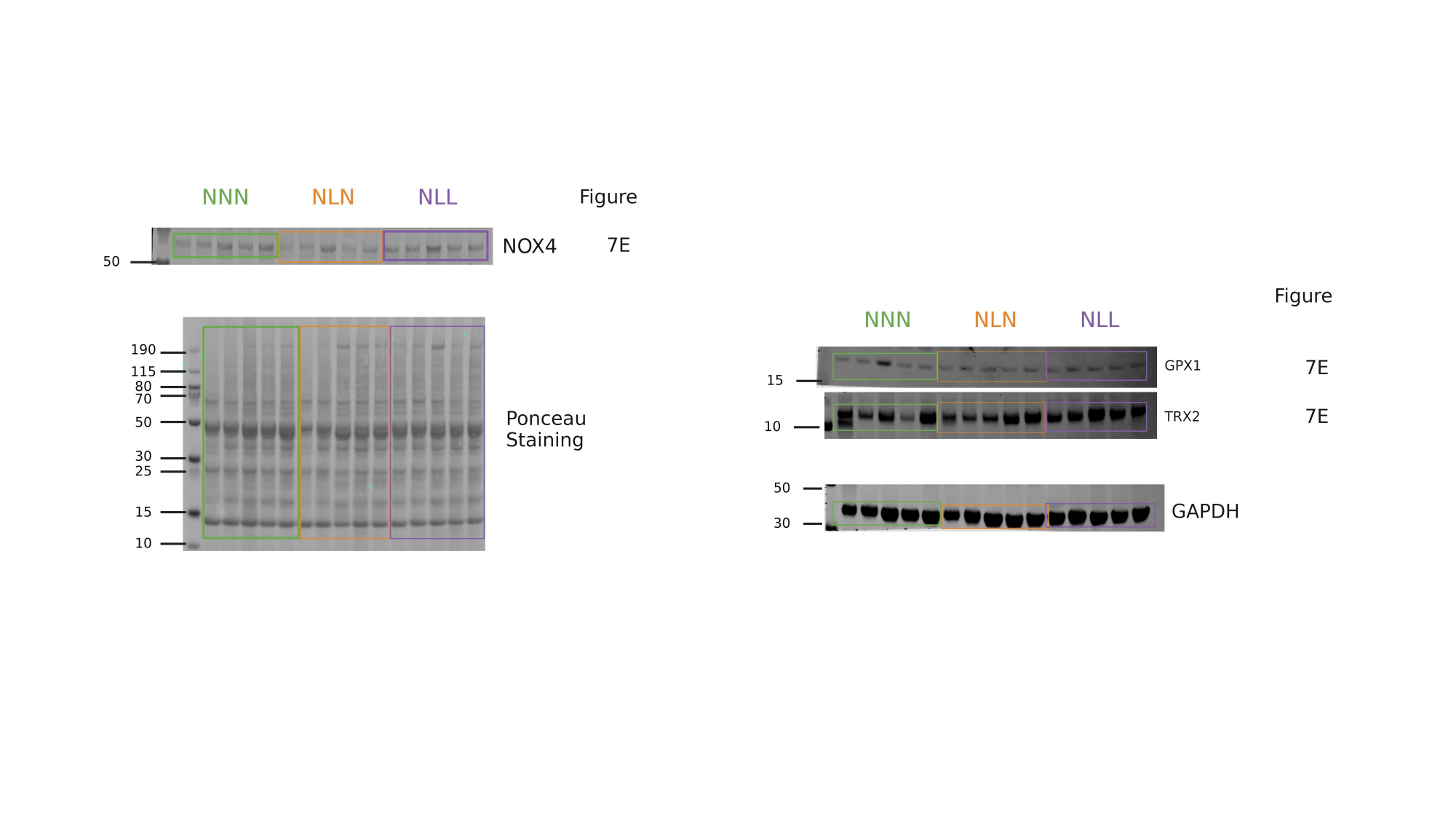

## Slide 6
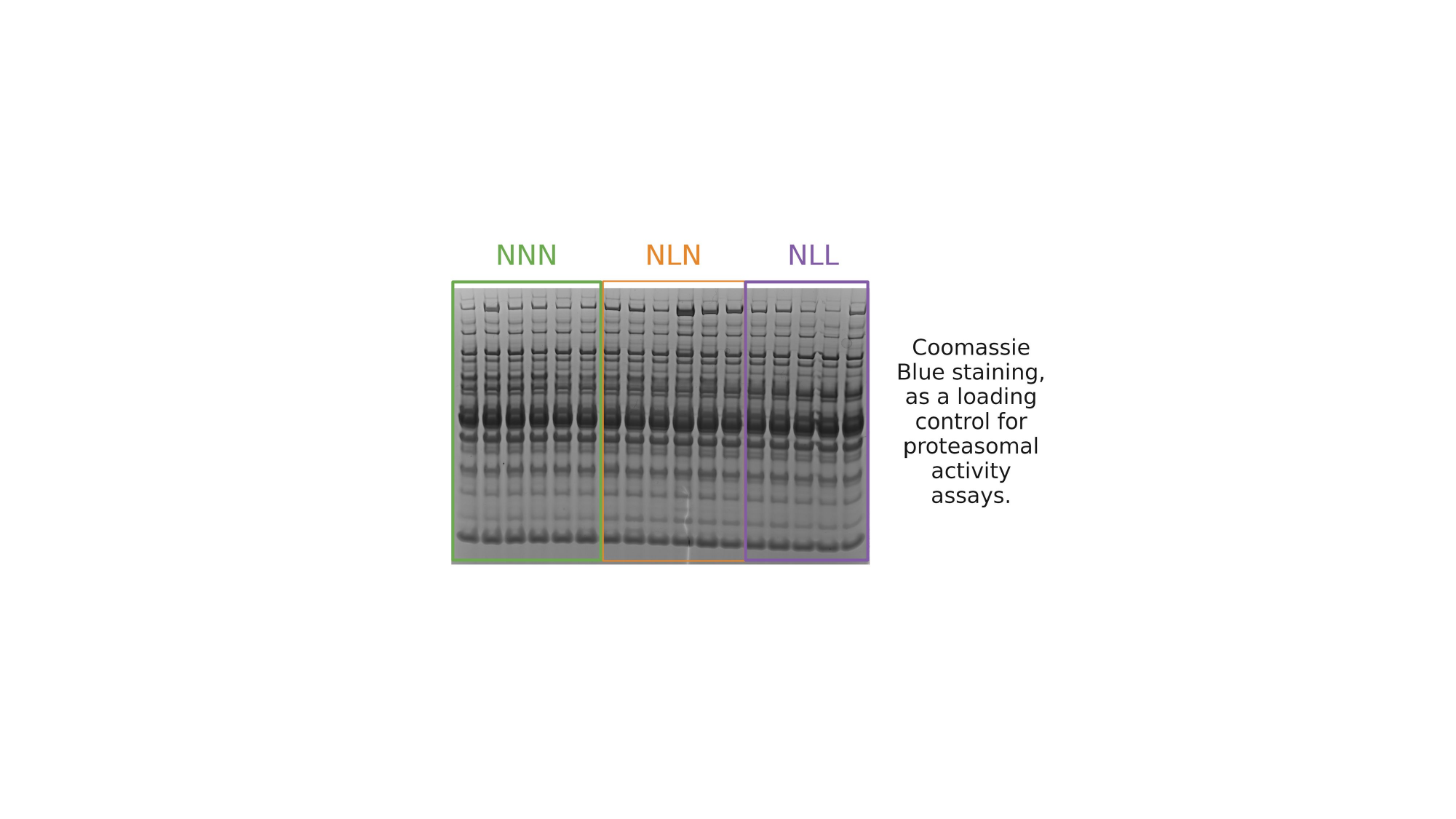

Supplement: Supplementary Material [file EMS202146-supplement-Supplementary_Material.zip › 1-s2.0-S0891584924006488-mmc2.pptx]
